# The complete plastid genome of *Thyrsostachys siamensis* (Poaceae, Bambusoideae)

**DOI:** 10.1080/23802359.2021.1934138

**Published:** 2021-05-27

**Authors:** Shu-Ling Tang, Jian-Hua Xie, Jia-Zhen Cai

**Affiliations:** College of Food Engineering, Zhangzhou Institute of Technology, Zhangzhou, China

**Keywords:** Bamboo, phylogeny, chloroplast genome

## Abstract

*Thyrsostachys* is oligotypic genus of Bambusinae, while its phylogenetic position had been unclear. Here, the complete plastid genome of the type species, *T. siamensis*, was sequenced and analyzed in this work. The complete genome is a typical quadripartite structure with 139,522 bp in length, comprising of a large single-copy region (LSC, 83,032 bp), a small single-copy region (SSC, 12,892 bp), and a pair of invert repeats regions (IR, 21,799 bp). The genome contains 138 genes, 89 protein-coding genes, 41 tRNA genes, and 8 rRNA genes. The GC content of genome was 38.9%. Phylogenetic analysis indicated *T. siamensis* was sister to *Dendrocalamus birmanicus* within Bambusinae.

The Bambusoideae is one of the largest subfamilies of the grass family (Poaceae) with approximately 1680 species (Liu et al. 2020). The phylogenetic classification of this subfamily recognizes three tribes, Arundinarieae, Olyreae and Bambuseae (Soreng et al. 2017). Among them, the Bambuseae is the largest tribe with approximately 970 species and divided into two major lineages: the neotropical woody bamboos (including Arthrostylidiinae, Chusqueinae and Guaduinae) and the paleotropical woody bamboos (including Bambusinae, Dinochloinae, Greslaniinae, Hickeliinae, Holttumochloinae, Melocanninae, Temburongiinae and Racemobambosinae) (Soreng et al. 2017; Zhou et al. 2017).

*Thyrsostachys* Gamble, belonging to Bambusinae, includes only two species, *T. siamensis* and *T. oliveri*, which are native to Yunnan, Myanmar, Thailand, Vietnam, and distributed from tropics to temperate regions of Asia (Li et al. 2006). Previous studies suggested that the position of *Thyrsostachys* was not clear. Based on the ITS, *GBSSI* and *trnL-F* sequences, the phylogenetic analysis of paleotropical woody bamboos showed that *Thyrsostachys* was monophyletic and sister to *Dendrocalamus*, *Gigantochloa*, *Oxytenanthera*, and *Neosinocalamus* (Yang et al. 2008). Based on the *GBSSI*, *psbA-trnH*, *rpl32-trnL* and *rps16* intron sequences, the phylogenetic analysis of *Bambusa* and its allies showed that *Thyrsostachys* was sister to *Melocalamus*, *Dendrocalamus*, *Gigantochloa*, and *Oxytenanthera* (Yang et al. 2010). Present study sequenced and assembled a complete plastid genome of the type species *T. siamensis* to investigate the genome feature and phylogenetic position.

The sample was acquired from Cangshan District, Fujian Province of China (26°38′N, 119°27′E), and the voucher specimen deposited in the Herbarium of the Forestry College of Fujian Agriculture and Forestry University (FJFC, specimen code Bamboo0910). Complete plastid genome of *Dendrocalamus latiflorus* (FJ970916) as reference, the paired-end reads were filtered by GetOrganelle pipe-line (https://github.com/Kinggerm/GetOrganelle) to get plastid-like reads (Jin et al. 2020), then the filtered reads were assembled by SPAdes version 3.10 (Bankevich et al. 2012). Then the final ‘fastg’ were filtered by the script of GetOrganelle to get pure plastid contigs, and the filtered De Brujin graphs were viewed and edited by Bandage (Wick et al. 2015). Assembled plastid genome annotation based on comparison with *D. latiflorus* by GENEIOUS v11.1.5 (Biomatters Ltd., Auckland, New Zealand) (Kearse et al. 2012). The matrix of 60 representative species of paleotropical woody bamboos were aligned using MAFFT v7.307 (Katoh and Standley 2013). Five genera of neotropical woody bamboos (*Chusquea circinata* of Chusqueinae; *Guadua angustifolia*, *Otatea glauca* and *O. reflexa* of Guaduinae; and *Merostachys* sp. and *Rhipidocladum pittieri* of Arthrostylidiinae) were selected as the outgroup according to Zhou et al. (2017) and Liu et al. (2020). The phylogenetic tree was constructed based on the complete plastid genomes by the maximum likelihood software IQ-TREE (Nguyen et al. 2015) and branch supports with the ultrafast bootstrap (Hoang et al. 2018). The analyses were performed using Phylosuite platform (Zhang et al. 2020).

The complete plastid genome of *T. siamensis* was a quadripartite structure with the length of 139,522 bp, contains a large single copy region (LSC) of 83,032 bp, a small single copy (SSC) region of 12,892 bp, and two inverted repeat (IR) regions of 21,799 bp. The genome possessed total 139 genes, including 89 protein-coding genes, 8 rRNA genes, and 41 tRNA genes. The overall GC content of the plastid genome was 38.9%, while the corresponding values of the LSC, SSC, and IR regions are 37.0%, 33.1%, and 44.2%, respectively.

The phylogenetic analysis of 65 Bambuseae plastomes showed that the paleotropical woody bamboos were grouped into three major clades, Melocanninae, Hickeliinae, and Bambusinae, with high support (Figure 1). The *T. siamensis* was sister to *Dendrocalamus birmanicus* within the Bambusinae clade. This newly reported plastid genome provides a good foundation for better understanding the generic relationships of subtribe Bambusinae.

**Figure 1.**
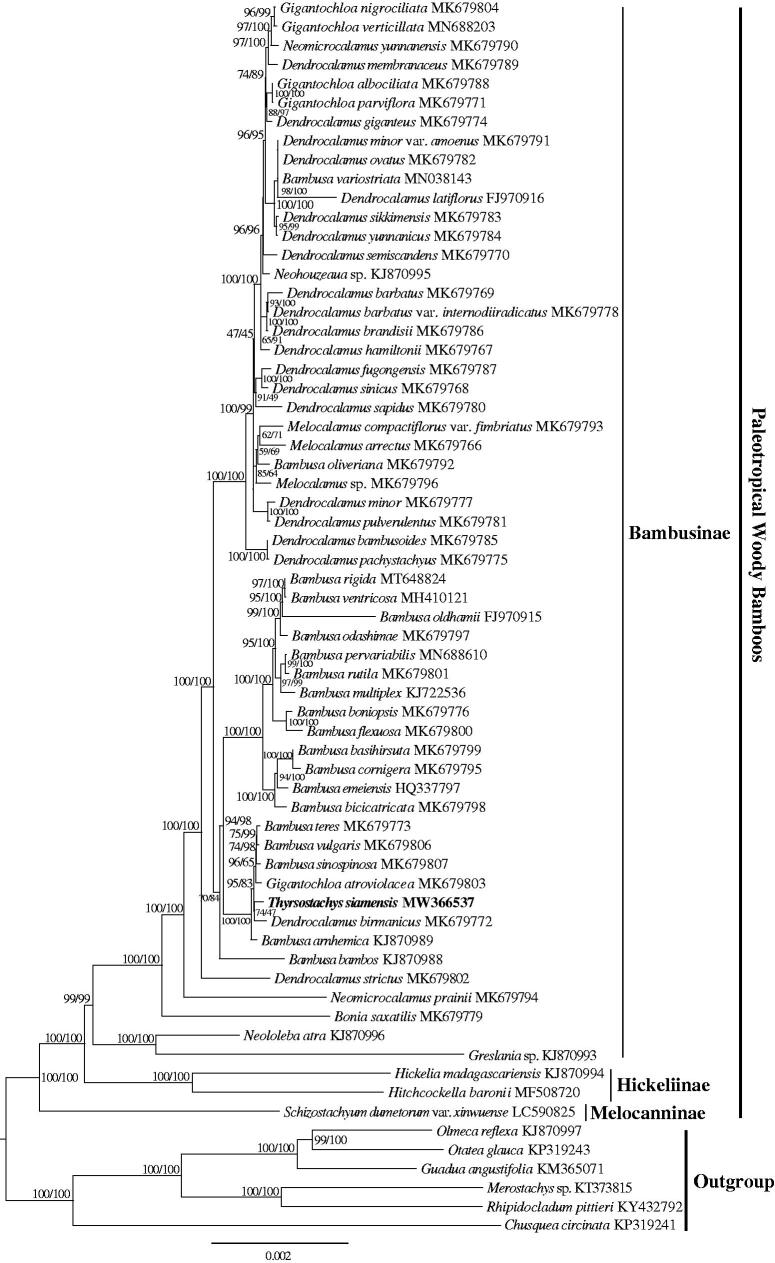
The Maximum-Likelihood (ML) tree based on the 65 plastid genomes of Bambuseae. Numbers near the nodes mean bootstrap support value (Standard bootstrap left and Ultrafast bootstrap right).

## Data Availability

The complete plastid genome of *Thyrsostachys siamensis* of this study is available in NCBI GenBank database (https://www.ncbi.nlm.nih.gov) with the accession code MW366537.
